# Validation of grading of non-invasive urothelial carcinoma by digital pathology for routine diagnosis

**DOI:** 10.1186/s12885-021-08698-4

**Published:** 2021-09-06

**Authors:** Richard Colling, Hayleigh Colling, Lisa Browning, Clare Verrill

**Affiliations:** 1 Nuffield Department of Surgical Sciences, University of Oxford, John Radcliffe Hospital, Oxford, OX3 9DU UK; 2 Department of Cellular Pathology, Oxford University Hospitals NHS Trust, John Radcliffe Hospital, Oxford, OX3 9DU UK; 3 NIHR Oxford Biomedical Research Centre, Oxford University Hospitals NHS Foundation Trust, Oxford, Oxfordshire UK

**Keywords:** Bladder, Urothelial, Carcinoma, Grade, Digital pathology

## Abstract

**Background:**

Pathological grading of non-invasive urothelial carcinoma has a direct impact upon management. This study evaluates the reproducibility of grading these tumours on glass slides and digital pathology.

**Methods:**

Forty eight non-invasive urothelial bladder carcinomas were graded by three uropathologists on glass and on a digital platform using the 1973 WHO and 2004 ISUP/WHO systems.

**Results:**

Consensus grades for glass and digital grading gave Cohen’s kappa scores of 0.78 (2004) and 0.82 (1973). Of 142 decisions made on the key therapeutic borderline of low grade versus high grade urothelial carcinoma (2004) by the three pathologists, 85% were in agreement. For the 1973 grading system, agreement overall was 90%.

**Conclusions:**

Agreement on grading on glass slide and digital screen assessment is similar or in some cases improved, suggesting at least non-inferiority of DP for grading of non-invasive urothelial carcinoma.

## Background

Most bladder tumours are urothelial carcinomas and around 70–80% of these are either non-invasive or early-invasive (superficial) [1]. Risk stratification based on morphological grading by pathologists is clinically useful for determining prognosis and follow-up management and therefore, histopathological grading confers significant clinical impact for patients. Despite this, little data exist on the reproducibility of these grading systems, especially for the increasing popularity and transitioning to digital pathology (DP) assessment of tumours [2, 3]. This is particularly important given that a number of potential pitfalls are already known in some areas of DP, where digital screen appearances can be challenging to identify or interpret. Most DP validation studies focus on overall diagnostic concordance rather than tumour grade specifically, however grading dysplasia and tumours is often identified as a source of discordance [4]. Recent reviews and guidelines highlight potential pitfalls of digitally grading atypia, including in urothelial cells [5–7]. This view has been supported by a number of validation studies that have identified grade discrepancies in the small number of urothelial carcinomas included [8–11]. The true extent of this problem in urological cancers, and how this relates to background intra and inter-observer variation, is not known. The aim of this study is to evaluate the intra-observer and inter-observer variation in grading of non-invasive urothelial bladder carcinomas, comparing glass and digital reporting/assessment methodologies.

## Methods

Fifty consecutive bladder cases, including transurethral resections and biopsies, of non-invasive papillary urothelial carcinomas were selected from the 2019 digital archive for a departmental audit. A formal sample size calculation was not performed; a small set of representative cases were selected, in line with routine validation type studies for laboratory studies. Cases were graded by three specialist uropathologists. All pathologists had at least 12 months experience with DP and the laboratory scans all routine paraffin-embedded histology slides. All cases were graded both on a digital screen and on traditional glass slides. Cases were graded twice on glass and once via DP, with a washout period of at least 2-weeks between sessions. Glass slides were missing for two cases, which were then excluded. Slides were scanned with a × 40 objective using a Philips Ultra Fast Scanner and displayed on a high-resolution (either an Eizo MX242W or a Dell U2715H), calibrated (to a brightness of at least 270 cd/m^2^, gamma of 2.2, and white point at 7500 K) digital screen using the Philips IMS on Google Chrome. Both ISUP/WHO 2004 and WHO 1973 systems were used for grading. Agreement was compared using linear weighted Cohen’s kappa and Fleiss’ kappa. A group consensus grade (by best of three votes) was also used for comparisons. All three pathologists were blinded to the original reports, each other’s grading, and the grades from their own previous assessment sessions (although potential access was available). Cases that were given a diagnosis of papillary urothelial neoplasm of low malignant potential (PUNLMP) during the study, were excluded from the statistical analysis.

## Results

**Fig. 1:**
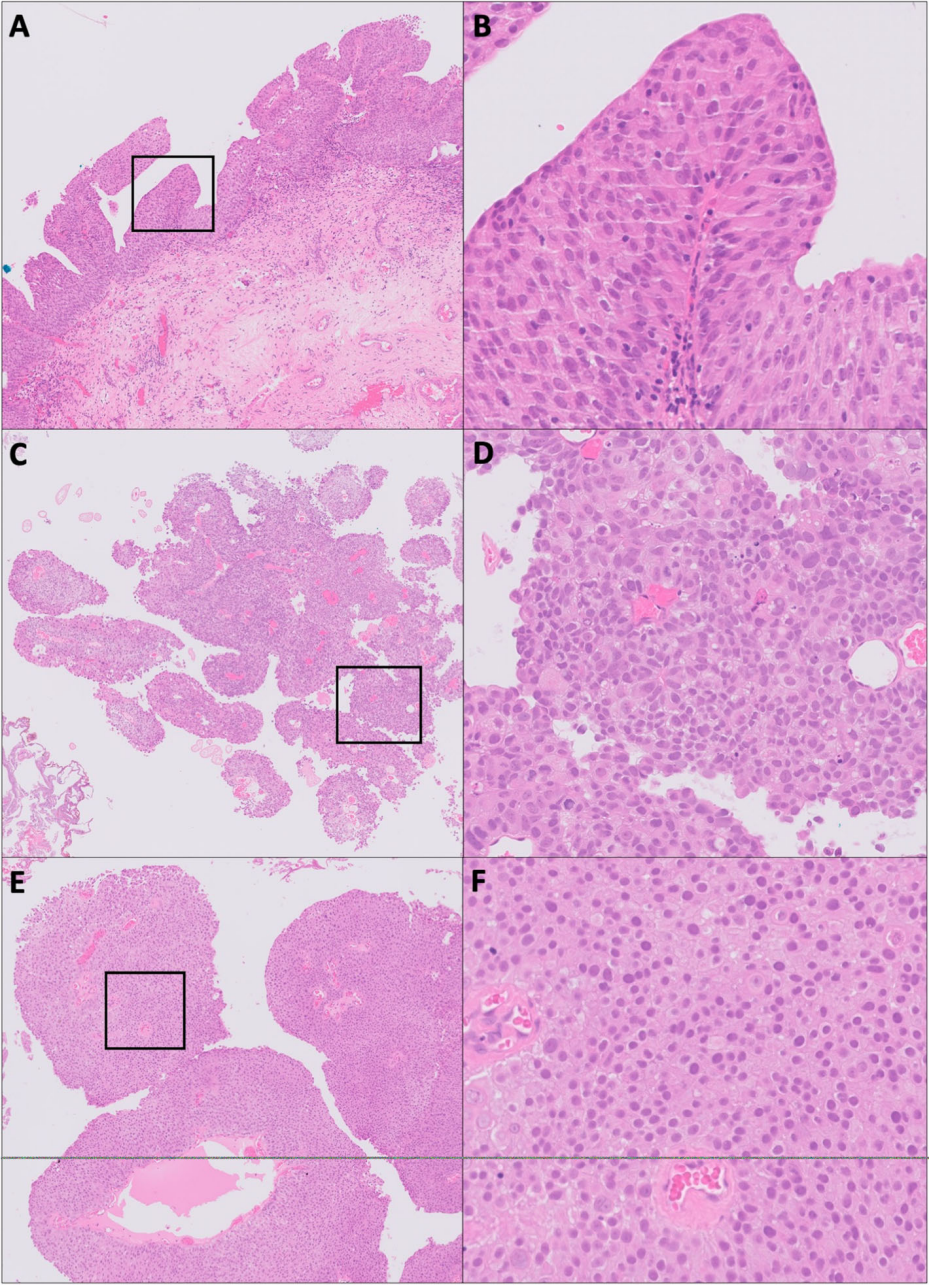
Example images of non-invasive urothelial carcinomas of the bladder graded in this study. Case 1, shown at low power (**A**) and the insert at high power (**B**), is an example of complete inter-observer agreement of a low grade, grade 2 tumour. There was also complete intra-observer agreement on subsequent grading sessions (glass and digital). Case 43 (**C**&**D**) is a high grade, grade 3 tumour with complete intra-observer and inter-observer agreement. Case 44 (**E**&**F**), is an example where pathologists B and C both downgraded the tumour from high grade on glass to low grade on digital assessment (pathologist A deemed this case high grade on both media)

The grades assigned to each case in the three separate grading sessions by each pathologist are given in Table 1. Examples of these are shown in Fig. 1. The kappa scores are summarised in Table 2.

**Table 1:** Grades assigned for each of the 48 cases in the study by each of the three pathologists. Grades were assigned on three occasions, separated by a washout period. Each of the grading sessions were completed on either digital pathology screen (once) or on glass slide (two separate sessions). Both the 1973 (grade 1, 2, 3) and 2004 (PUNLMP, low, high) WHO grading systems were applied for each case in all sessions. Pathologists were blinded throughout the study to the results of the original report, to the grades they gave at previous assessment sessions within the study, and to the grades given by the other pathologists in the study. Discrepancies between the digital reporting and first glass reporting are highlighted in red

Case	Pathologist A						Pathologist B						Pathologist C					
	Digital	Digital	1st Glass	1st Glass	2nd Glass	2nd Glass	Digital	Digital	1st Glass	1st Glass	2nd Glass	2nd Glass	Digital	Digital	1st Glass	1st Glass	2nd Glass	2nd Glass
1	L	2	L	2	L	2	L	2	L	2	L	2	L	2	L	2	L	2
2	L	2	L	2	L	2	L	2	L	2	H	2	L	2	L	2	L	2
3	L	2	L	2	L	2	L	2	L	2	H	2	L	2	L	2	H	2
4	H	3	H	3	H	3	H	2	H	2	H	2	H	2	H	2	H	3
5	L	1	L	2	L	2	L	2	L	2	L	2	L	2	L	2	L	2
6	H	2	H	3	H	3	L	2	L	2	H	2	L	2	H	2	H	2
7	L	2	L	2	L	2	L	2	L	2	L	2	L	2	L	2	L	2
8	L	2	L	2	L	2	L	2	L	2	L	2	L	2	L	2	L	2
9	L	2	L	2	L	2	L	2	H	2	H	3	L	2	L	2	H	2
10	L	2	H	2	L	2	L	1	L	1	L	2	L	2	L	2	L	2
11	H	3	H	3	H	2	H	2	H	2	H	3	H	3	H	3	H	2
12	H	2	L	2	L	2	L	2	H	2	H	2	H	2	H	2	L	2
13	H	3	H	3	H	3	H	2	H	2	H	2	H	3	H	3	H	3
14	L	2	L	2	L	2	L	2	L	2	L	2	L	2	H	2	L	2
15	L	2	L	2	L	2	L	2	L	2	L	1	L	2	L	2	L	2
16	L	2	H	3	H	3	H	3	H	3	H	3	H	3	H	3	H	3
17	L	2	L	2	L	2	L	2	L	2	L	2	L	2	L	2	L	2
18	H	3	H	3	H	3	H	2	H	3	H	3	H	3	H	3	H	3
19	H	2	H	2	L	2	L	2	L	2	L	2	H	2	L	2	L	2
20	L	2	L	2	L	2	L	2	L	2	L	2	L	2	H	2	L	2
21	H	2	L	2	L	2	L	2	L	2	L	2	H	2	L	2	L	2
22	H	2	H	2	L	2	L	2	L	2	L	2	H	2	L	2	L	2
23	L	2	L	2	L	1	L	2	L	2	L	2	L	2	L	2	L	2
24	L	2	L	2	L	2	L	2	L	2	L	2	L	2	L	2	L	2
25	H	3	H	3	H	3	H	3	H	3	H	3	H	3	H	3	H	3
26	H	3	L	2	H	3	H	3	H	2	H	3	H	3	H	3	H	3
27	L	1	L	2	L	2	L	2	L	2	L	2	L	2	L	2	L	2
28	H	3	H	3	L	2	H	2	H	2	H	2	H	2	L	2	H	2
29	H	2	H	2	L	2	L	2	L	2	L	2	L	2	L	2	L	2
30	L	2	L	2	L	1	L	2	L	2	L	2	L	2	L	2	H	2
31	H	3	H	3	H	2	L	2	L	2	L	2	H	2	H	3	H	3
32	L	2	L	2	L	2	L	2	L	2	L	2	L	2	L	2	L	2
33	PUNLMP	N/A	PUNLMP	N/A	PUNLMP	N/A	L	2	L	2	L	2	L	2	L	2	L	2
34	L	2	L	2	L	2	L	2	L	2	L	2	L	2	L	2	L	2
35	H	3	H	3	L	2	H	2	H	2	H	3	L	2	H	2	H	2
36	H	3	H	3	H	3	H	3	H	3	H	3	H	2	H	3	H	2
37	L	2	L	2	L	2	L	2	L	2	L	2	L	2	L	2	L	2
38	H	3	H	3	H	3	H	3	H	2	H	3	H	3	H	3	H	2
39	L	2	L	2	L	2	H	2	L	2	L	2	L	2	H	2	H	2
40	L	2	L	2	L	2	L	2	L	2	L	2	L	2	L	2	L	2
41	L	2	H	2	PUNLMP	N/A	L	2	L	2	L	2	L	2	L	2	L	2
42	PUNLMP	N/A	PUNLMP	N/A	PUNLMP	N/A	L	2	L	2	L	2	L	2	L	2	L	2
43	H	3	H	3	H	3	H	3	H	3	H	3	H	3	H	3	H	3
44	H	3	H	3	H	2	L	2	H	2	H	3	L	2	H	2	H	2
45	H	3	H	3	H	3	H	2	H	2	H	3	L	2	H	3	H	3
46	H	3	H	3	H	3	H	3	H	3	H	3	H	3	H	3	H	3
47	H	3	H	2	H	3	H	2	H	2	H	3	H	2	H	3	H	3
48	H	2	H	3	L	2	L	2	L	2	H	2	L	2	L	2	L	2

*L* Low grade, *H* High grade, *PUNLMP* Papillary urothelial neoplasm of low malignant potential

**Table 2:** Cohen’s and Fleiss’ kappa scores for grading of non-invasive urothelial carcinoma of the bladder. Individual pathologist grades and pathologist consensus grades compared (top) between 1st and 2nd glass slide grading sessions and between glass slide (1st grading) and digital pathology grading. Agreement between all three pathologists (below) are given for both glass slide grading sessions and digital pathology grading. Both 1973 and 2004 grading systems compared

	WHO 1973 1st vs. 2nd glass grading (Cohen’s kappa, linear weighted)	WHO 2004 1st vs. 2nd glass grading (Cohen’s kappa, linear weighted)	WHO 1973 Glass vs. digital grading (Cohen’s kappa, unweighted)	WHO 2004 Glass vs. digital grading (Cohen’s kappa, unweighted)
**Pathologist A**	0.55	0.64	0.70	0.74
**Pathologist B**	0.46	0.83	0.77	0.82
**Pathologist C**	0.78	0.70	0.77	0.53
**Consensus**	0.71	0.83	0.82	0.78
	**1973** (Fleiss’ kappa)	**2004** (Fleiss’ kappa)		
**Agreement of all three pathologists on 1st glass grading**	0.49	0.56		
**Agreement of all three pathologists on 2nd glass grading**	0.56	0.70		
**Agreement of all three pathologists on digital grading**	0.44	0.61		

For the 2004 grading system, the number of cases that were in agreement between digital and 1st glass grading for pathologist A was 40/46 (87%), for pathologist B was 44/48 (92%), and for pathologist C was 37/48 (77%), with overall 121/142 (85%) grades in agreement. Of the 21 discrepancies, 13 cases (62%) of the cases deemed high grade on glass were downgraded to low grade on digital, whereas the remaining eight (38%) low grade cases on glass were deemed high grade on digital. A similar trend towards digital downgrading was seen in the 1973 grading system. Here, the number of cases in agreement for pathologist A was 39/46 (85%), for pathologist B was 45/48 (94%), and for pathologist C was 44/48 (92%), with overall 128/142 (90%). Of the 14 discrepancies, six (43%) were deemed grade 2 on glass with four (28%) upgraded to grade 3 and two (14%) downgraded to grade 1 on glass, and eight (57%) were deemed grade 3 on glass and downgraded to grade 2 on digital – 10 cases were therefore downgraded on digital (71% of the 14 cases).

## Discussion

The impact of grading on clinical management of patients with superficial bladder cancer is significant. The presence of high grade morphology will often up-stratify a tumour with patients sometimes offered mitomycin C, Bacille Calmette-Guérin (BCG) therapy, or even surgery. It is imperative then that pathologists can reliably grade these tumours, including using increasingly popular DP systems.

Grading of atypia/dysplasia is a requirement for in situ, non-invasive, and invasive neoplasms associated with tumours arising at many sites and thus is applicable beyond just urothelial neoplasia. Some authors have expressed concerns that grading using low power views on a digital screen may pose a risk for missing focal areas of more high-grade disease [5–9]. If this is the case, the issue would be more pertinent to tumours such as urothelial carcinoma (based on highest grade area, even if small or focal) than for other tumours where grading may be an overall appearance. However, it is arguable that the same may be true with traditional glass microscopy.

In this study three pathologists who specialise in reporting urological specimens retrospectively graded a set of 48 non-invasive urothelial carcinomas on three separate occasions. Cases were graded twice on glass (to assess intra-pathologist consistency) and once on a digital screen as a comparison. All grading sessions were carried out with blinding and washout periods. Although a relatively small sample was used, this type of approach is in keeping with standard practice when validating new laboratory equipment and larger sample sizes (over 50) for agreement studies for a very specific context do not usually improve the statistical analysis.

The agreement of all three pathologists on a digital screen grading was moderate, with slightly better performance for the 2004 grading system – as might be expected for a system with fewer categories. This overall level of agreement is not unexpected and is in keeping with data that have been reported in bladder cancers before [12]. For example, a reproducibility study of grading Ta/T1 bladder cancers in 2014 found kappa scores for the agreement between seven pathologists ranging from 0.68 to 0.70 [12]. Similar problems are also reported at other tissue sites [13–15]. Specifically on DP systems, studies have identified high discrepancy rates in interpretation of urothelial biopsies when compared with glass slide interpretation [16] and grading urothelial atypia is cited as a common problem [5–8]. In this study however, no obvious trend in agreement of the three pathologists was seen on glass versus digital, suggesting that digital grading was as good as glass grading.

Intra-observer agreement of pathologists (agreement of pathologists with themselves) was generally better than agreement between (inter-observer) pathologists, regardless of modality. This is probably to be expected for subjective grading systems and so, as has been suggested by some authors, intra-observer agreement may be a more reliable indicator of the reproducibility of DP than inter-observer agreement [4, 17]. In keeping with that view, this study suggests that, overall, DP is non-inferior for grading non-invasive bladder cancer.

Consensus grades (the grade agreed by at least two pathologists) produced largely the highest kappa scores in the study, suggesting that double reporting may also be a useful and safe way of checking grading for potentially high stakes cases in routine practice. With DP, this is increasingly easy as cases can be electronically shared with colleagues at the click of a mouse.

As expected, most of the disagreements (on glass and digitally) were a difference of only one grade either way, and most differences would have no, or very little, impact on patient management. There were cases where all three pathologists agreed on the grade (both grading systems) for all grading sessions (Cases 1, 7, 8, 17, 24, 25, 32, 34, 37, 40, 43, 46, see Table 1), but these were only 12 occasions (25%) and tended to be low grade, grade 2 cases, arguably a middle default grade.

Low grade versus high grade (WHO 2004) and grade 2 versus grade 3 (WHO 1973) are key therapeutic thresholds. In this study, in 87% (2004) and 90% (1973) of cases the grades between pathologists were in agreement on digital and glass assessment, with a slight tendency to undergrade/downgrade on digital. Similar levels of agreement were found in a recent systematic review, which showed a 92.4% agreement between digital and glass *diagnosis*, but overall diagnosis would probably have less potential for subjective inter-observer variation than grading [18]. The mild tendency to undergrade on digital, could be explained by the observation that pathologists might be inclined to use a lower magnification digital view and miss areas of high grade tumour. Other possible explanations are difficulties with rendering of nuclear detail on digital images, poor focusing, the effect of file compression artefact, and the limited dynamic range of the whole slide image. It is also possible that this trend may not be reproduced in larger studies. Difficulties with diagnosis and grading of atypia / dysplasia on the digital microscope is nonetheless a recurrent theme in the literature and is a potential pitfall for the new digital pathologist [6]. The need for confirming borderline cases on both digital and glass and also asking for second opinions/double reporting when in doubt is re-iterated by the findings in this study.

## Conclusions

In this study we have shown that agreement for grading non-invasive bladder tumours on glass slide and digital screen assessment is similar, or in some cases improved by digital reporting. The data suggest that digital reporting of grade in these tumours is at least non-inferior and we have outlined how others can adopt and validate similar techniques in their centres.

## Data Availability

All data generated or analysed during this study are included in this published article.

## Abbreviations

BCG: Bacille Calmette-Guérin
DP: Digital pathology
H: High grade
ISUP: International society of urological pathology
L: Low grade
PUNLMP: Papillary urothelial neoplasm of low malignant potential
WHO: World Heath Organisation
